# Subcritical Water Hydrolysis of Comb Pen Shell (*Atrina pectinata*) Edible Parts to Produce High-Value Amino Acid Products

**DOI:** 10.3390/md20060357

**Published:** 2022-05-27

**Authors:** Byung-Soo Chun, Seung-Chan Lee, Truc-Cong Ho, Jean-Bosco Micomyiza, Jin-Seok Park, David Nkurunziza, Hee-Jeong Lee

**Affiliations:** 1 Department of Food Science and Technology, Pukyong National University, 45 Yongso-ro, Nam-gu, Busan 48513, Korea; bschun@pknu.ac.kr (B.-S.C.); sclee0762@gmail.com (S.-C.L.); hocongtruc@mku.edu.vn (T.-C.H.); micobosco@gmail.com (J.-B.M.); newjin1931@pknu.ac.kr (J.-S.P.); davidnkurunziza145@gmail.com (D.N.); 2 PL MICROMED Co., Ltd., 1F, 15-5, Yangju 3-gil, Yangsan-si 50620, Korea; 3 Department of Food and Nutrition, Kyungsung University, 309 Suyeong-ro, Nam-gu, Busan 48434, Korea

**Keywords:** subcritical water hydrolysis, *Atrina pectinate* edible parts, amino acids profile, antioxidant activity, antihypertensive activity

## Abstract

*Artina pectinata* (Comb pen shell, CPS) is a high-protein source that contains a variety of essential amino acids. Subcritical water hydrolysis (SWH) was used to recover amino acids from the posterior adductor muscle (PAM), anterior adductor muscle (ADM), and mantle. The temperatures ranged from 120 °C to 200 °C, and the pressure and time of hydrolysis were 3 MPa and 30 min, respectively. Further characterization of the hydrolysates was performed to ascertain amino acid profiles and biofunctional properties. The hydrolysates contained more free amino acids than the untreated samples. Antioxidant activity of treated samples increased as SW temperatures increased. At 200 °C, those inhibiting ACE had a maximum antihypertensive activity of 200 °C in 1% PAM, ADM, and mantle with 85.85 ± 0.67, 84.55 ± 0.18, and 82.15 ± 0.85%, respectively, compared to 97.57 ± 0.67% in 1% standard captopril. Perhaps the most significant finding was the predominance of taurine in the three parts following SW treatment at 120 °C. The hydrolysates may be of considerable interest for use in food or energy drinks. SWH demonstrates efficacy in recovering amino acids, particularly taurine, from edible parts of *A. pectinata.*

## 1. Introduction

*Atrina pectinata*, also known as comb pen shell (CPS), is a large wedge-shaped bivalve in the Pinnidae family. CPS is found throughout the Indo-West Pacific, ranging from southern Africa to Malaysia and New Zealand, north to Japan, and south to New South Wales. CPS is primarily found in Korea near the south seashore of Yeojaman, the west seashore of Boryeong, and Taean [1], and is harvested in large quantities each year. Furthermore, CPS powder contains approximately 60% proteins, 9.6% carbohydrates, 17.2% lipids, and 10% ash, according to our previous study [2]. Apart from direct consumption, proteins from CPS can be recovered and concentrated for use in a variety of other products, such as food supplements or energy drinks.

Different techniques, such as organic solvent extraction and enzymatic treatment, have been conventionally applied to extract valuable compounds from different sources. However, these processes have a number of disadvantages, including the formation of undesirable by-products, lower yields, solvent residue in the extract, solvent toxicity, degradation of the target compounds, and time consumption [3,4]. 

Furthermore, the potential application of new green technology based on supercritical fluids, namely subcritical water extraction (SWE) or subcritical water hydrolysis (SWH), is a crucial technological development. SWH is capable of extracting a wide variety of bioactive compounds [5] using water as a solvent without compromising the extracted products’ quality [6]. The physicochemical properties of water vary with temperature, allowing it to dissolve hydrophobic compounds at near-critical conditions (P and T below the critical point) [7]. This means that when water is subjected to a temperature between 100 and 374 °C and a high enough pressure to maintain the liquid state—known as subcritical water (SW)—it can extract compounds such as conventionally used solvents [8]. Water has a high dielectric constant (ε) under normal conditions due to its extensive hydrogen bonding structure, making it ineffective for extracting non-polar or organic compounds. In SW, the hydrogen bonds are broken by the temperature and pressure, therefore changing these properties [9]. This property makes water the safest solvent because it does not require the solvent to be removed from the final products, as needed in other solvents [10]. 

Previously, we demonstrated that SWH was an effective method for obtaining amino acids from the viscera of CPS [2]. However, no consideration has been made for the recovery of these high-value compounds using SW from separated components of this abundant protein source. Therefore, in this study, the CPS was divided into three edible components: PAM (posterior adductor muscle), ADM (anterior adductor muscle), and mantle. Then, in an SWH system, each component was hydrolyzed at different SW temperatures. Additionally, the hydrolysates were characterized in terms of amino acid composition and biological activity.

## 2. Results and Discussion

### 2.1. Proximate Compositions

The proximate compositions depicted in Table 1 indicated a predominance of crude proteins. Although the protein content of the three components is identical, it is significantly greater than the 60.70% reported in the previous study for CPS powder [2]. Protein content might unequally contribute to different edible parts of the comb pen shell and might be dominant in the three parts. Carbohydrate content varied between 11.69% and 25.21%, with PAM having the highest content, followed by ADM and mantle. The carbohydrate content of the three components is greater than that of the CPS powder (9.60%). In contrast, the crude lipids and ash contents, are lower than those previously reported in our work (17.20% and 9.98%, respectively) [2]. The crude protein content analysis revealed that the three components are of interest for obtaining essential peptides and amino acids.

### 2.2. Hydrolysis Efficiency and the Changes in Molecular Weight (MW)

**Figure 1:**
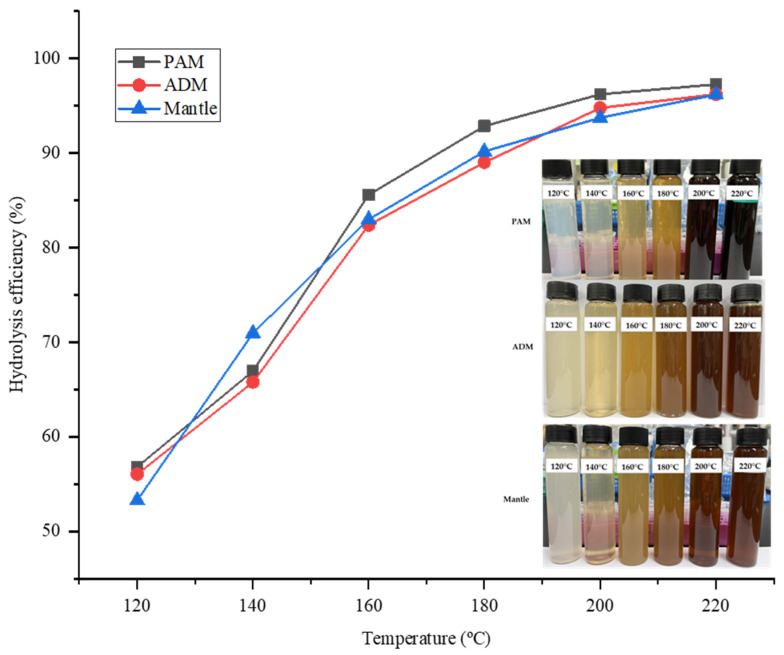
SWH efficiency for different parts of *Atrina pectinata* (PAM: posterior adductor muscle; ADM: anterior adductor muscle).

The hydrolysis efficiency (HE) is a critical parameter that indicates the amount of sample that has been hydrolyzed following treatment with SHW. In all parts, HE increased proportionately with temperature. While the temperature of the water rapidly increased from 120 °C to 180 °C, the rate of increase slowed between 180 °C and 220 °C. As illustrated in Figure 1, the highest HE value was 98.65% at 220 °C in all three parts of the CPS. This result indicated that the majority of the three components were effectively hydrolyzed by SW treatment, owing to the decomposition of large proteins and carbohydrates into low molecular weight molecules such as peptides, amino acids, and reducing sugars. Additionally, because water’s polarity is inversely proportional to its temperature, it increases the solubility of hydrophobic proteins in SW.

The changes in molecular weights of PAM, ADM, and mantle at different temperatures in SWH are presented in Table 2. After being treated at 120 °C, MW of the components in PAM, ADM, and the mantle is between 816,008 and 1010,109 Da. The MW rapidly decreased to 1292, 1264, and 1324 Da for PAM, ADM, and mantle, respectively, as the temperatures of the SWH reached 220 °C. These results agreed with the explanation that at elevated temperatures, peptide bonds of protein molecules are broken down, resulting in the formation of smaller molecules of soluble protein or amino acids [11]. 

### 2.3. Content of Total Protein and Reducing Sugar, and Color Changes of the Hydrolysates

**Figure 2:**
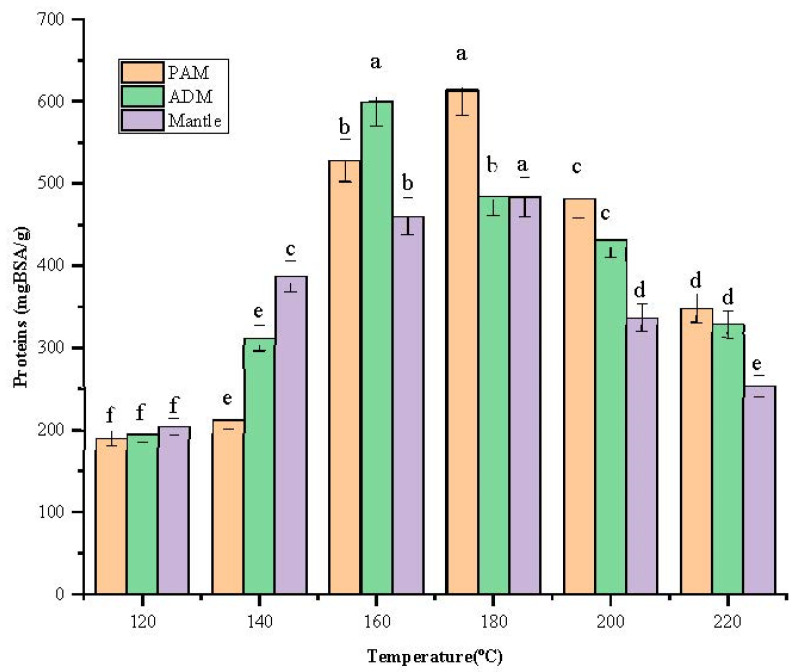
Effect of varying subcritical water treatment temperatures on the total protein content of different parts of *Atrina pectinata* (PAM: posterior adductor muscle; ADM: anterior adductor muscle).

After being treated in SW, the total protein content of the three parts increased remarkably with increasing temperature from 120 °C to 180 °C, as shown in Figure 2. In addition, the content peak was 613.7 ± 2.67 mgBSA/g at 180 °C for PAM. At 160 °C, the highest protein content was found in ADM and mantle, at 600.2 ± 5.26 mgBSA/g and 484.2 ± 3.81 mgBSA/g, respectively.

Different letters of the bars in the same color indicate significant differences (*p* < 0.05). These findings indicate that these three components have distinct structural characteristics, necessitating the use of distinct temperatures to degrade the cellular structural components and release the proteins. The decreasing trend occurred beyond 180 °C because biomolecules might be degraded at elevated temperatures. According to a previous report, temperatures above 180 °C resulted in protein degradation into amino acids and organic acids [11], lowering the overall protein content. Additionally, the Maillard reaction can occur at elevated temperatures in the presence of amino acids and reducing sugar [12].

**Figure 3:**
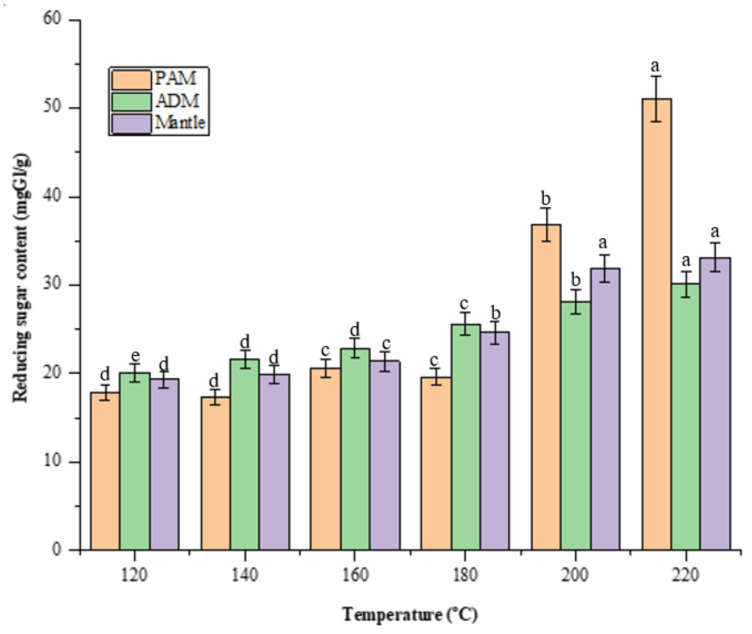
Effect of varying subcritical water treatment temperatures on reducing sugar content of different parts of *Atrina pectinata* (PAM: posterior adductor muscle; ADM: anterior adductor muscle). Different letters of the bars in the same color indicate significant differences (*p* < 0.05).

Screening for reducing sugars is necessary because they play a critical role in the Maillard reaction, which has antioxidant and antihypertensive properties [13]. Figure 3 illustrates the reducing sugars formed from total sugar during the high-temperature treatment of SW conditions. The increase in reducing sugar is a result of carbohydrates being decomposed into their monosaccharides. PAM treated at 200 °C contained the most reducing sugars (51.1 ± 0.98 mgGl/dried sample), significantly more than ADM and mantle, which contained 30.1 ± 0.6 and 33.1 ± 0.62 mgGl/dried sample, respectively. A previous study discovered a high reducing sugar content in dried squid muscle hydrolysates at 220 °C, which decreased as the SW temperature increased to 280 °C [14].

The changes in the color of PAM, ADM, and mantle are presented in Table 3. In general, the increase in darkness of the three samples was observed as the temperatures of the SWH increased from 120 to 220 °C. This result is also consistent with the previous data in the same range of temperatures [2]. The darkest color was observed in the hydrolysate of PAM that was treated at 200 and 220 °C. The high content of protein and reducing sugar (presented in Figure 2 and Figure 3) of the sample might lead to the formation of more browning products from Maillard reactions as compared to the rest. 

### 2.4. Bound and Free Amino Acid Content

**Figure 4:**
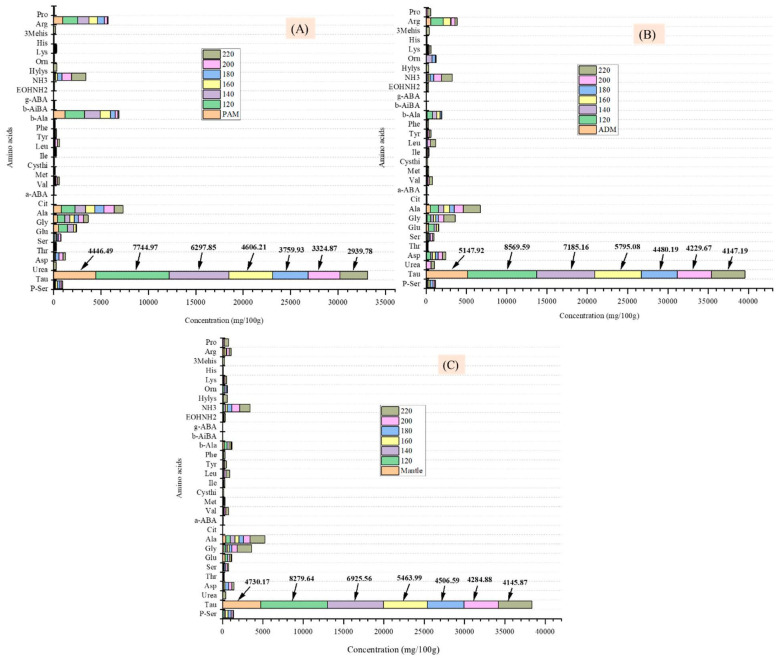
Effect of varying subcritical water treatment temperatures on free amino acid content of different parts of *Atrina pectinata* (PAM: posterior adductor muscle (**A**); ADM: anterior adductor muscle (**B**), and mantle (**C**).

As shown in Table 4, nineteen bound amino acids were identified and quantified in the three components of the CPS. The amino acids glutamic acid, aspartic acid, arginine, alanine, and leucine were the most abundant. Glutamic acid was found in the highest concentrations, at 78.6 mg/g in the PAM, 90.96 mg/g in the ADM, and 78.5 mg/g in the mantle. Glutamic acid levels were also found to be elevated in CPS viscera, as previously reported [2]. As shown in Figure 4, after treating all parts of the CPS in SW at various temperatures, the number of free amino acids in the hydrolysates increased significantly compared to the untreated samples. From 180 °C and above, the amino acid concentration began to decrease due to the degradation of these components to form carbonic acids, amines, and other organic acids [15,16], as well as the role of glutamic acid in the Maillard reaction at elevated temperatures [17]. Additionally, the results indicate that increasing the SW temperature promotes the recovery of other essential amino acids such as alanine, arginine, and so on. This could be because of the increased dissolving capacity of water caused by a low water polarity and dielectric constant power. These findings corroborated those of a previous study in which abalone was treated in SW at temperatures ranging from 110 to 230 °C [18]. 

### 2.5. Taurine Content

The significantly high amount of taurine in all three parts of CPS after treatment in SW was perhaps one of the most important findings of the study. Taurine is a critical amino acid found in the brain, reproductive organ cells, the heart, and the retina of humans, as well as in meat and seafood. This amino acid possesses a variety of biofunctional properties, including antioxidant activity, the ability to scavenge reactive oxygen species, protection against oxidative stress to organs, anti-inflammatory properties, and the ability to emulsify and digest lipids [19]. 

The taurine content of untreated PAM, ADM, and mantle was between 4446.49 and 5147.92 mg/100 g (Figure 4), which was significantly greater than the taurine content of some other marine resources reported in the previous study [19]. When the three components were treated in SW to 120 °C, the taurine content was nearly doubled (7744.97, 8569.59, and 8279.64 mg/100 g in PAM, ADM, and mantle, respectively) (Figure 4A–C). Taurine was primarily an inert biochemical intermediate between methionine and cysteine [20]. At 220 °C, the taurine content of the three components decreased gradually to 2939.78, 4147.19, and 4145.87 mg/100 g, respectively. The proportional decrease in content with increasing temperature is due to this compound’s thermal degradation. The presence of a significant amount of taurine in the hydrolysates of three edible parts of CPS at 120 °C generates considerable interest for food and energy drink applications, as there may be no deterioration of bioactive components at this commonly used temperature for food processing.

### 2.6. Antioxidant Capacity of the Hydrolysates

**Figure 5:**
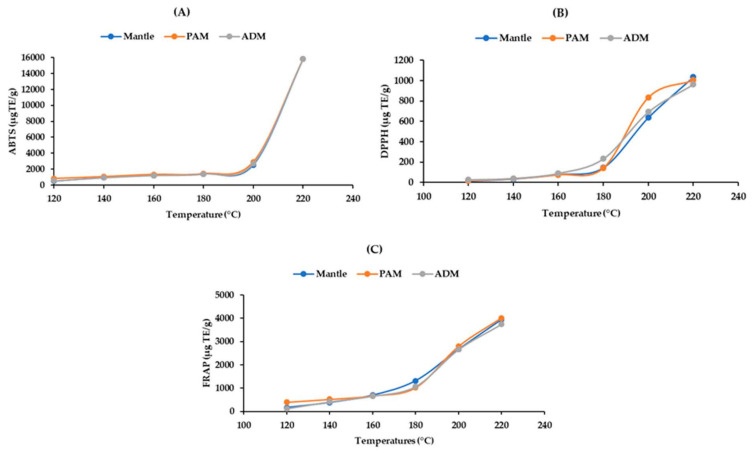
Effect of varying subcritical water treatment temperatures on the antioxidant activity of different parts of *Atrina pectinata* (PAM: posterior adductor muscle; ADM: anterior adductor muscle). (**A**)—ABTS assay; (**B**)—DPPH assay; (**C**)—FRAP assay.

Antioxidant capacity is a property of proteins and their derivatives, such as peptides and amino acids. These compounds contain both hydrophobic and hydrophilic molecules. As a result, ABTS is an appropriate method for determining these components’ antioxidant activity. On the other hand, FRAP and DPPH, which are widely used due to their simplicity and convenience, can only be used to evaluate hydrophilic antioxidants. Thus, it is sensible that ABTS would have higher values than the other two assays. At temperatures below 200 °C, the hydrolysates’ ABTS radical scavenging activity increased slightly; however, it increased to 15,786 ± 1.59 μg TE/g in all ADM, PAM, and mantle at 220 °C (Figure 5A). The DPPH and FRAP results revealed a similar pattern of antioxidant capacity (Figure 5B,C). The increase in antioxidant capacity of the three edible parts of CPS following SW hydrolysis could be attributed to the formation of smaller peptides and free amino acids. Taurine, an amino acid with a high antioxidant capacity, may contribute significantly to radical scavenging due to its high concentration [19]. Additionally, the Maillard reaction’s end products have antioxidant properties [21]. Furthermore, our findings are consistent with those of the previous study [14]. 

### 2.7. Effect on the Antihypertensive Activity

Hypertension (high blood pressure) is a global health problem that is associated with stroke and cardiovascular disease mortality. Recent research indicates that hypertension can be treated by inhibiting the angiotensin-converting enzyme (ACE) [22]. The use of a natural antihypertensive compound is a superior alternative to synthetic drugs because it minimizes or eliminates any adverse effects associated with those synthetic drugs [23]. After being hydrolyzed in SW, all three edible parts of CPS demonstrated significant ACE-inhibitory activity. The activity increased as the SW temperature was increased from 120 to 200 °C and decreased as the treatment temperature was increased above 200 °C. 

**Figure 6:**
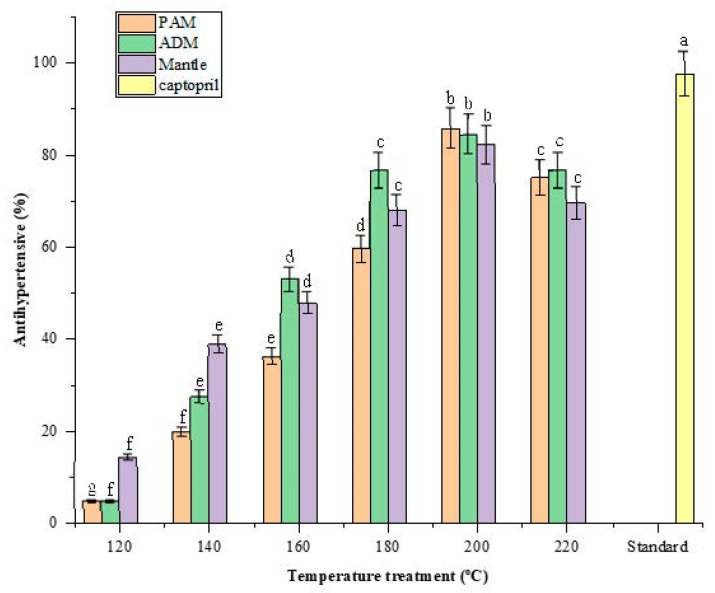
Effect of varying subcritical water treatment temperatures on the antihypertensive activity of different parts of *Atrina pectinata* (PAM: posterior adductor muscle; ADM: anterior adductor muscle). Captopril was used as a standard antihypertensive drug for comparison. Different letters of the bars in the same color indicate significant differences (*p* < 0.05).

The maximum inhibitory effect of ACE was approximately 85% in 1% PAM, ADM, and mantle, compared to 97.57 ± 0.67% in 1% standard captopril as presented in Figure 6. According to previous reports, peptides derived from food proteins from plants and animals exhibit ACE-inhibitory activity. The inhibition of ACE by food-derived peptides is most likely due to the competitive inhibition of the enzyme catalytic sites by peptides or amino acids [24]. Taurine may also have an antihypertensive effect, which may be a result of its effect on the central nervous system [25]. Another study found that Maillard reaction products possessed this biofunctional property as well. Although the mechanism remains unknown, it is known that ACE is a zinc-dependent enzyme and that Maillard reactions involving melanoidins may be related to their metal-chelating properties [13]. 

## 3. Materials and Methods

### 3.1. Materials

The comb pen shell was kindly provided by Hallyeosusan, Gyeongsangnam-do, and Sacheon. It was then separated into three components, PAM, ADM, and mantle, before being cleaned with water and freeze-dried at a temperature of –110 °C. Dry samples were ground and sieved to obtain fine particles and stored at a temperature of 70 °C for subsequent use. All chemicals used in this study were HPLC or analytical grade.

### 3.2. Approximate Compositions Analysis

The approximate composition was determined using the same method as described previously [5]. In summary, ash content was determined by calcining a 0.5 g sample at 600 °C for 6 h; crude lipid was recovered using hexane as the extraction solvent and a Soxhlet system for 24 h; crude protein was determined by measuring total nitrogen using a Kjeldahl digester and multiplying it by 6.25 as the nitrogen conversion factor, and carbohydrate content was determined by subtracting all of the above components from the total ash. All experiments were replicated three times.

### 3.3. Subcritical Water Extraction

**Figure 7:**
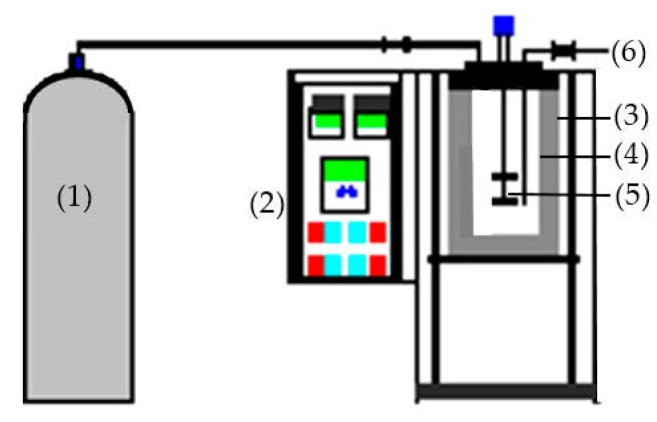
Diagram of the SWH apparatus; (1)—Nitrogen cylinder, (2)—Control board, (3)—Heating jacket, (4)—Reactor, (5)—Agitator, (6)—Sampling valve.

The SHW was carried out using 1000 mL batch-type subcritical water (Figure 7). A sample (20 g) and 600 mL distilled water were added to the reactor. Each batch was heated to 120, 140, 160, 180, 200, and 220 °C; pressure and hydrolysis time were set to 3 MPa and 30 min, respectively. Filtration of the hydrolysates was performed using Whatman filter paper, and the extract was stored at −70 °C for subsequent use. The hydrolysis efficiency (HE) was determined using the following equation:

HE (%) = (1 − S_h_/S_r_) × 100

where S_h_ denotes the weight of the residue (g), and S_r_ denotes the weight of the sample (g).

### 3.4. Reducing Sugar 

The reducing sugar content of the hydrolysates was determined using the previously described DNS colorimetric method [26]. In order to prepare DNS (3,5-dinitrosalicylic) solution, 30 g sodium hydroxide tartrate or Rochelle salt was dissolved in 80 mL of 0.5 N NaOH solution and diluted to 100 mL with distilled water. In order to determine the reducing sugar, a sample (1 mL) was added to 4 mL DNS, heated to 96 °C for 5 min, cooled to room temperature for 20 min, and the absorbance a 540 nm was measured. The reducing sugar content was expressed as mg of glucose equivalent per 100 g dried sample (mg Gl/g).

### 3.5. Total Protein Content

The extract (0.6 mL) was added to 3 mL of Lowry’s solution, thoroughly mixed for a few seconds, and allowed to stand for 20 min in the dark. Folin–Ciocalteu solution (1 N, 0.3 mL) was added to the mixture and incubated in the dark for 35 min. At 750 nm, readings were taken using a microplate reader. The results are expressed in milligrams of bovine serum albumin (BSA) per gram of dried sample.

### 3.6. Antioxidant Capacity

In order to prepare a stock solution for the ABTS assay, equivalent amounts of 7 mM ABTS+ and 2.45 mM potassium persulfate were mixed and subjected to a 16-h dark reaction at room temperature. One milliliter of ABTS+ stock solution was combined with60 mL MeOH solution. The absorbance at 734 nm was determined and adjusted to 0.7 ± 0.02. The sample was diluted with MeOH (1:3, *v*/*v*), and 100 µL of the supernatant was combined with 3.9 mL of ABTS+ solution for 6 min in the dark. Afterward, absorbance was measured at 734 nm. In the DPPH assay, the sample was diluted with MeOH (1:3, *v*/*v*), 100 µL of the supernatant was mixed with 3.9 mL of 0.2 mM DPPH ethanolic solution, and a dark reaction was performed at room temperature for 30 min. Afterward, absorbance was measured at 517 nm. Next, to determine the antioxidant activity using FRAP assay, a FRAP solution was prepared by mixing 300 mM acetate buffer, 10 mM TPTZ in 40 mM HCl, and 20 mM iron (III) chloride in a ratio of 10:1:1 (*v*/*v*). The sample was then diluted with MeOH (1:3, *v*/*v*), and 0.3 mL of the supernatant was mixed with 3 mL of FRAP solution, followed by reaction at room temperature for 4 min in the dark place. Afterward, absorbance was measured at 593 nm.

### 3.7. Antihypertensive Activity

Antihypertensive activity of the hydrolysates was determined according to the previous study [5] using the ACE kit-WST manual (Dojindo Molecular Technologies, Inc. Rockville, MD, USA). The absorbance was measured at 450 nm using a microplate reader, and captopril (1%) was used as a standard. The hydrolysates’ ACE inhibitory activity was expressed as a percentage (%).

### 3.8. Analysis of Bound and Free Amino Acids

The sample was pretreated prior to analyzing the total amino acid content. Briefly, 60 mg of sample were melted in 6N HCl before being hydrolyzed at 110 °C for 22 h. The samples were then vacuum-dried before melting in 0.02 N HCl 10 mL followed by filtering through a 0.2 µm syringe filter. In the preparation of samples to analyze free amino acid, sample (3 g) was melted in 70% EtOH 30 mL. The solution was shaken for 1 h followed by keeping for 10 min. Subsequently, the solution was centrifuged at 15,000 revolutions per minute for 15 min to obtain the supernatant. Then, 30% aqueous EtOH was added to completely submerge, and the procedure was repeated three times. The supernatant was vacuum-dried and then melted in 20 mL 0.02 N HCl. Total and free amino acids were determined using an amino acid analyzer L-8800 (Hitachi, Tokyo, Japan) equipped with an ion-exchange column (4.6 mm × 60 mm), column oven temperature 57–62 °C, and reaction coil temperature 135 °C. Channels 1 and 2 had UV-Vis detector wavelengths of 570 and 440 nm, respectively. The injection volume was 20 l, and pump 1 and pump 2 operated at flow rates of 0.4 and 0.34 mL/min, respectively.

### 3.9. Measurement of Color

The color of the hydrolysates was determined using a reflectance colorimeter (Lovibond RT series, Tintometer Ltd., Amesbury, UK). The values from the CIELAB color space were expressed as three-dimensional values, L* (Lightness), a* (Redness) and b* (Yellowness).

### 3.10. Gel Permeation Chromatography (GPC)

The molecular weight was determined using gel permeation chromatography (Dionex Ultimate 3000, Sunnyvale, CA, USA) with Ultrahydrogel column and RI detector. Before analysis, the freeze-dried sample was diluted in deionized water to form a 1% solution, and the injection volume of 50 μL was kept constant. The determination of molecular weight was based on the calibration curve of the pullulan standard (342–803,000 Da).

### 3.11. Statistical Analysis

SPSS version 23 for Windows (IBM, Chicago, IL, USA) was used for statistical analysis. The results were expressed as the mean ±SD (*n* =3), with a significance level of *p* < 0.05 considered statistically significant.

## 4. Conclusions

In the present study, three edible parts (PAM, ADM, and mantle) of the CPS were hydrolyzed in SW. The hydrolysis efficiency increased proportionately as the treatment temperature increased from 120 °C to 220 °C. The hydrolysates contained more amino acids than the untreated samples. Therefore, their antioxidant activity, as measured by ABTS, DPPH, and FRAP, increased as well. The antihypertensive effect through ACE-inhibition activity of the hydrolysates reached its peak at 200 °C. These exhibit superior biofunctional properties due to the presence of derived peptides and amino acids, as well as Maillard reaction end products. More importantly, all hydrolysates of the three edible parts treated at 120 °C contained almost twice the amount of taurine found in untreated samples and the highest amount of taurine found in hydrolysates treated at other SW temperatures. This is the first finding that may attract considerable attention in food and energy drink applications, as the hydrolysates may be safe for direct consumption at this temperature. While additional research is necessary to confirm safety, the current findings suggest a novel approach to valorizing the edible parts of CPS.

## Figures and Tables

**Table 1:** Proximate compositions of *A. pectinata* parts (PAM: posterior adductor muscle; ADM: anterior adductor muscle).

Compositions	PAM (%)	ADM (%)	Mantle (%)
Crude lipid	2.72 ± 0.80 ^a^	3.72 ± 1.33 ^b^	3.68 ± 0.49 ^b^
Crude protein	64.70 ± 1.24 ^a^	66.80 ± 1.08 ^a^	64.90 ± 1.11 ^a^
Moisture	2.02 ± 0.13 ^a^	1.91 ± 0.09 ^a^	3.08 ± 0.13 ^b^
Ash	5.35 ± 0.02 ^a^	8.98 ± 0.15 ^b^	16.65 ± 0.32 ^c^
Carbohydrate	25.21 ± 0.55 ^a^	18.59 ± 0.66 ^b^	11.69 ± 0.51 ^c^

Different superscripts in the same row indicate the statistical difference.

**Table 2:** Changes in molecular weights of the samples at different temperatures of SWH.

Samples	Average Molecular Weight (Da)					
	120 °C	140 °C	160 °C	180 °C	200 °C	220 °C
PAM	993,368	679,527	367,247	172,730	7169	1292
ADM	816,004	551,550	323,440	138,939	1652	1264
Mantle	1,010,109	475,506	237,344	114,841	1826	1324

**Table 3:** Color of the hydrolysates.

Temperatures (°C)	PAM			ADM			Mantle		
	L *	a *	b *	L *	a *	b *	L *	a *	b *
120	60.41 ± 1.9 ^a^	1.08 ± 0.15 ^c^	6.65 ± 0.11 ^d^	55.76 ± 1.11 ^b^	1.01 ± 0.08 ^c^	14.17 ± 0.5 ^b^	58.87 ± 2.53 ^a^	0.33 ± 0.06 ^d^	7.56 ± 0.15 ^e^
140	62.58 ± 1.12 ^a^	−0.10 ± 0.03 ^f^	8.61 ± 0.13 ^c^	63.27 ± 1.21 ^a^	−0.57 ± 0.09 ^d^	9.95 ± 0.16 ^d^	61.93 ± 1.82 ^a^	−0.76 ± 0.08 ^e^	11.51 ± 0.89 ^c^
160	57.72 ± 0.86 ^b^	0.44 ± 0.08 ^e^	17.47 ± 1.21 ^b^	55.33 ± 1.50 ^b^	0.90 ± 0.92 ^c^	19.17 ± 0.27 ^a^	49.22 ± 1.81 ^b^	1.22 ± 0.55 ^c^	15.34 ± 0.99 ^b^
180	53.10 ± 1.11 ^c^	2.94 ± 0.53 ^b^	24.41 ± 1.23 ^a^	44.67 ± 1.27 ^c^	4.96 ± 0.06 ^b^	18.46 ± 0.94 ^a^	44.22 ± 1.35 ^c^	4.48 ± 0.04 ^b^	17.71 ± 1.51 ^a^
200	31.99 ± 0.96 ^d^	6.16 ± 0.95 ^a^	2.15 ± 0.10 ^e^	38.01 ± 0.98 ^d^	9.17 ± 1.16 ^a^	12.16 ± 0.41 ^c^	38.64 ± 1.12 ^d^	9.53 ± 0.08 ^a^	8.79 ± 0.12 ^d^
220	30.56 ± 0.82 ^d^	0.69 ± 0.09 ^d^	−0.39 ± 0.03 ^f^	34.93 ± 0.88 ^e^	9.55 ± 0.55 ^a^	7.49 ± 0.19 ^e^	35.67 ± 1.42 ^e^	8.55 ± 0.89 ^a^	7.31 ± 1.31 ^e^

Different superscript letters in the same column indicate the significant difference (*p* < 0.05). *: CIELAB color scale.

**Table 4:** Bound amino acid compositions of different parts of *Atrina pectinata* (PAM: posterior adductor muscle; ADM: anterior adductor muscle).

Amino Acid Compositions	Content (mg/g)		
	PAM	ADM	Mantle
Aspartic acid	53.96	65.34	53.70
Threonine	23.05	27.77	24.67
Serine	26.38	33.46	29.48
Glutamic acid	78.69	90.96	78.51
Glycine	22.67	38.08	55.52
Alanine	46.82	53.23	42.59
Cysteine	3.97	5.88	5.63
Valine	13.61	17.26	15.64
Methionine	14.00	11.34	11.99
Isoleucine	11.06	12.83	10.97
Leucine	44.80	52.67	37.98
Tyrosine	16.89	20.63	15.97
Phenylalanine	29.84	19.19	17.74
Lysine	39.35	42.14	31.62
NH3	6.83	8.52	7.36
Histidine	8.83	9.27	7.37
Arginine	49.36	57.38	44.27
Hypro	0.00	5.78	13.02
Proline	15.81	25.57	32.13
